# Prevalence and Disparities in Tobacco Product Use Among American Indians/Alaska Natives — United States, 2010–2015

**DOI:** 10.15585/mmwr.mm6650a2

**Published:** 2017-12-22

**Authors:** Satomi Odani, Brian S. Armour, Corinne M. Graffunder, Bridgette E. Garrett, Israel T. Agaku

**Affiliations:** 1 Office on Smoking and Health, National Center for Chronic Disease Prevention and Health Promotion, CDC.

An overarching goal of *Healthy People 2020* is to achieve health equity, eliminate disparities, and improve health among all groups.* Although significant progress has been made in reducing overall commercial tobacco product use,^†^ disparities persist, with American Indians or Alaska Natives (AI/ANs) having one of the highest prevalences of cigarette smoking among all racial/ethnic groups (*1*,*2*). Variations in cigarette smoking among AI/ANs have been documented by sex and geographic location (*3*), but not by other sociodemographic characteristics. Furthermore, few data exist on use of tobacco products other than cigarettes among AI/ANs (*4*). CDC analyzed self-reported current (past 30-day) use of five tobacco product types among AI/AN adults from the 2010–2015 National Survey on Drug Use and Health (NSDUH); results were compared with six other racial/ethnic groups (Hispanic; non-Hispanic white [white]; non-Hispanic black [black]; non-Hispanic Native Hawaiian or other Pacific Islander [NHOPI]; non-Hispanic Asian [Asian]; and non-Hispanic multirace [multirace]). Prevalence of current tobacco product use was significantly higher among AI/ANs than among non-AI/ANs combined for any tobacco product, cigarettes, roll-your-own tobacco, pipes, and smokeless tobacco. Among AI/ANs, prevalence of current use of any tobacco product was higher among males, persons aged 18–25 years, those with less than a high school diploma, those with annual family income <$20,000, those who lived below the federal poverty level, and those who were never married. Addressing the social determinants of health and providing evidence-based, population-level, and culturally appropriate tobacco control interventions could help reduce tobacco product use and eliminate disparities in tobacco product use among AI/ANs (*1*).

NSDUH is an annual, national survey of the civilian, noninstitutionalized U.S. population aged ≥12 years (*4*). The analyses in this report were restricted to persons aged ≥18 years. Because of the limited sample size of AI/ANs, data were pooled across six NSDUH waves (2010–2015) to increase precision of estimates; pooled sample sizes were 3,655 for AI/AN adults and 235,262 for non-AI/AN adults.^§^ Annual response rates averaged 65.4% among all respondents. The AI/AN population included persons who identified AI/AN as their only race/ethnicity on the survey. Non-AI/AN populations comprised whites; blacks; NHOPIs; Asians; multiracial persons; and Hispanics. Current tobacco product use was defined as past 30-day use of the following tobacco products: cigarettes; cigars (big cigars, cigarillos, or little cigars); roll-your-own tobacco; pipes; and smokeless tobacco (chewing tobacco, snuff, dip, and snus).^¶^ Current users of any tobacco product** were persons who reported past 30-day use of one or more of the assessed tobacco product types.

Data were weighted to adjust for nonresponse and to yield nationally representative estimates. Prevalence was calculated overall and by sex, age group (18–25 years, 26–34 years, 35–49 years, and ≥50 years), education (less than a high school diploma, high school graduate, some college, college graduate), annual family income (<$20,000, $20,000–$49,999, $50,000–$74,999, and ≥$75,000), poverty,^††^ and marital status; prevalence estimates with relative standard errors ≥30% were suppressed. Non-AI/AN adults were used as comparison groups, both as a single combined group comprising the six other racial/ethnic groups and as individual racial/ethnic groups. Among AI/ANs, disparities in tobacco product use within sociodemographic subgroups were calculated using prevalence ratios (PRs) with 95% confidence intervals, with the group with the lowest prevalence of any tobacco use serving as the referent. Statistical comparisons were performed with Chi-square tests, with statistical significance defined as p<0.05.

During 2010–2015, prevalence among AI/ANs was significantly higher than that among non-AI/ANs combined for current use of any tobacco product (43.3% versus 27.7%, respectively); cigarettes (37.3% versus 23.0%); roll-your-own tobacco (7.1% versus 3.5%), pipes (1.9% versus 0.9%) and smokeless tobacco (6.6% versus 3.5%) (Table 1). With the exception of persons with a college degree or higher, current use of any tobacco product, cigarettes, and smokeless tobacco were all significantly higher among AI/ANs than their combined non-AI/AN counterparts within all subgroups. For current cigar smoking prevalence, a significant difference between AI/ANs and non-AI/ANs combined was seen among persons aged 35–49 years. Current use prevalence of roll-your-own tobacco was significantly higher among AI/ANs, compared with their combined non-AI/AN counterparts, for all subgroups except persons with less than a high school diploma; living in poverty; and widowed, divorced, or separated. Compared with their combined non-AI/AN counterparts, current pipe smoking prevalence was significantly higher among AI/AN males, as well as among persons aged 35–49 years; those with annual family income <$20,000; living in poverty; and who were never married (all p<0.05).

**TABLE 1:** Current use of tobacco products among AI/AN and non-AI/AN adults aged ≥18 years, overall and by sociodemographic and socioeconomic characteristics — National Survey on Drug Use and Health, 2010–2015

Characteristic	Any tobacco product*	Cigarettes	Cigars (big cigars/ cigarillos/little cigars)	Roll-your-own tobacco	Pipe	Smokeless tobacco (snuff/dip/chewing/snus)
	% (95% CI)	% (95% CI)	% (95% CI)	% (95% CI)	% (95% CI)	% (95% CI)
**AI/AN adults (N = 3,655)**						
**All**	43.3 (40.1–46.5)^†^	37.3 (34.2–40.3)^†^	5.9 (4.7–7.2)	7.1 (5.7–8.4)^†^	1.9 (1.1–2.8)^†^	6.6 (5.5–7.8)^†^
**Sex**						
Male	49.7 (44.9–54.5)^†^	39.8 (35.3–44.3)^†^	9.6 (7.2–12.0)	8.6 (6.4–10.8)^†^	2.7 (1.2–4.2)^†^	11.7 (9.4–13.9)^†^
Female	37.8 (33.6–42.0)^†^	35.1 (31.0–39.2)^†^	2.7 (1.7–3.8)	5.7 (3.9–7.5)^†^	–^§^	2.3 (1.5–3.1)^†^
**Age group (yrs)**						
18–25	55.6 (51.6–59.7)^†^	47.3 (43.2–51.5)^†^	12.2 (9.4–14.9)	9.7 (7.3–12.1)^†^	2.3 (1.1–3.6)	10.1 (7.8–12.4)^†^
26–34	53.0 (46.9–59.1)^†^	47.8 (41.7–53.9)^†^	8.4 (4.8–12.0)	11.9 (7.3–16.6)^†^	–^§^	9.1 (5.7–12.5)^†^
35–49	49.7 (44.2–55.3)^†^	41.8 (36.4–47.2)^†^	7.2 (4.0–10.4)^†^	6.1 (4.2–8.1)^†^	2.4 (1.1–3.6)^†^	7.8 (5.5–10.0)^†^
≥50	29.6 (23.8–35.4)^†^	25.4 (19.9–31.0)^†^	–^§^	4.5 (2.2–6.8)^†^	–^§^	3.3 (1.7–4.9)^†^
**Education**						
<High school	49.8 (42.8–56.8)^†^	45.1 (38.3–51.9)^†^	7.2 (4.3–10.2)	9.7 (6.4–13.1)	–^§^	7.6 (4.9–10.3)^†^
High school	45.3 (40.2–50.4)^†^	39.7 (34.7–44.7)^†^	4.8 (3.1–6.5)	8.3 (5.7–10.9)^†^	1.1 (0.5–1.7)	7.5 (5.6–9.3)^†^
Some college	43.5 (37.6–49.4)^†^	36.5 (31.0–42.0)^†^	5.7 (3.6–7.8)	5 0.0(3.2–6.7)^†^	–^§^	6.3 (4.2–8.4)^†^
≥College	21.0 (13.9–28.1)	13.1 (7.6–18.5)	–^§^	–^§^	–^§^	2.5 (1.1–3.9)
**Annual family income**						
<$20,000	50.3 (44.7–55.9)^†^	45.8 (40.3–51.4)^†^	6.9 (4.6–9.2)	10.7 (7.8–13.6)^†^	2.7 (1.3–4.2)^†^	6.9 (4.8–8.9)^†^
$20,000–$49,999	41.2 (36.1–46.3)^†^	36.8 (32.0–41.7)^†^	5.0 (3.4–6.6)	6.5 (4.3–8.7)^†^	0.5 (0.2–0.8)	6.2 (4.5–7.9)^†^
$50,000–$74,999	40.6 (32.4–48.8)^†^	30.2 (23.1–37.3)^†^	3.4 (0.9–6.0)	4.2 (1.9–6.4)^†^	–^§^	7.3 (3.9–10.6)^†^
≥$75,000	32.4 (25.2–39.6)^†^	21.0 (15.4–26.6)^†^	8.3 (3.5–13.1)	–^§^	–^§^	6.7 (3.7–9.7)^†^
Poverty level**						
Poverty	51.3 (45.6–57.0)^†^	46.8 (41.2–52.5)^†^	7.6 (5.1–10.1)	10.5 (7.5–13.4)	2.6 (1.1–4.2)^†^	7.2 (5.0–9.4)^†^
Up to 2x threshold	43.5 (37.8–49.2)^†^	38.2 (32.7–43.7)^†^	4.4 (2.7–6.0)	7.3 (4.7–9.9)^†^	0.8 (0.4–1.3)	6.6 (4.6–8.6)^†^
>2x threshold	36.0 (31.1–40.9)^†^	28.1 (23.8–32.4)^†^	5.6 (3.5–7.7)	3.9 (2.2–5.5)^†^	–^§^	6.1 (4.3–7.8)^†^
**Marital status**						
Married	37.9 (33.0–42.8)^†^	31.4 (26.8–36.0)^†^	4.5 (2.7–6.2)	4.3 (2.3–6.3)^†^	–^§^	5.5 (3.7–7.4)^†^
Widowed/Divorced/Separated	40.9 (33.7–48.1)^†^	36.8 (29.8–43.7)^†^	–^§^	6.0 (3.5–8.5)	–^§^	5.0 (3.0–7.1)^†^
Never married	50.5 (45.8–55.2)^†^	43.4 (38.9–47.9)^†^	9.8 (7.3–12.3)	10.6 (8.0–13.3)^†^	2.5 (1.1–3.9)^†^	9.0 (7.0–10.9)^†^
**Non-AI/AN (N = 235,262)**						
**All**	27.7 (27.4–27.9)	23.0 (22.7–23.2)	5.1 (5.0–5.3)	3.5 (3.4–3.6)	0.9 (0.8–0.9)	3.5 (3.4–3.6)
**Sex**						
Male	34.3 (33.9–34.8)	25.8 (25.4–26.2)	8.5 (8.3–8.8)	4.4 (4.2–4.6)	1.5 (1.4–1.6)	6.7 (6.5–7.0)
Female	21.5 (21.1–21.8)	20.3 (20.0–20.7)	2.0 (1.9–2.1)	2.6 (2.5–2.7)	0.3 (0.3–0.3)	0.4 (0.4–0.5)
**Age group (yrs)**						
18–25	37.2 (36.8–37.6)	30.7 (30.4–31.1)	10.3 (10.0–10.6)	5.0 (4.8–5.2)	1.9 (1.8–2.0)	5.7 (5.5–5.9)
26–34	36.9 (36.3–37.6)	31.6 (31.0–32.3)	7.3 (7.0–7.7)	4.4 (4.1–4.7)	0.9 (0.8–1.0)	4.6 (4.3–4.9)
35–49	30.1 (29.5–30.6)	24.8 (24.4–25.3)	4.6 (4.4–4.9)	3.6 (3.4–3.8)	0.5 (0.5–0.6)	4.2 (4–4.5.0)
≥50	19.7 (19.3–20.2)	16.2 (15.7–16.6)	2.9 (2.7–3.1)	2.5 (2.3–2.7)	0.7 (0.6–0.8)	1.9 (1.7–2.1)
**Education**						
<High school	36.0 (35.2–36.8)	31.8 (31.1–32.6)	6.0 (5.7–6.4)	7.3 (6.8–7.7)	1.4 (1.2–1.6)	4.2 (3.9–4.6)
High school	33.5 (32.9–34.0)	28.7 (28.2–29.3)	5.2 (4.9–5.4)	4.4 (4.2–4.6)	0.9 (0.8–1.0)	4.4 (4.1–4.6)
Some college	29.9 (29.4–30.5)	24.8 (24.3–25.3)	5.8 (5.5–6.0)	3.2 (3.0–3.4)	0.9 (0.8–0.9)	3.7 (3.5–3.9)
≥College	16.0 (15.6–16.5)	11.5 (11.2–11.9)	4.1 (3.9–4.3)	1.0 (0.9–1.2)	0.6 (0.5–0.7)	2.1 (1.9–2.2)
**Annual family income**						
<$20,000	37.5 (36.8–38.2)	33.6 (32.9–34.2)	6.7 (6.4–7.0)	7.8 (7.5–8.1)	1.5 (1.3–1.6)	3.3 (3.1–3.6)
$20,000–$49,999	30.3 (29.8–30.8)	26.3 (25.8–26.8)	4.8 (4.6–5.0)	3.8 (3.6–4.0)	0.9 (0.8–1.0)	3.3 (3.1–3.5)
$50,000–$74,999	25.2 (24.5–25.9)	20.5 (19.9–21.1)	4.4 (4.1–4.7)	2.3 (2.1–2.5)	0.8 (0.6–0.9)	3.7 (3.4–3.9)
≥$75,000	20.9 (20.4–21.4)	15.1 (14.7–15.5)	5.0 (4.7–5.2)	1.3 (1.2–1.4)	0.6 (0.5–0.7)	3.7 (3.5–3.9)
**Poverty level** ^¶^						
Poverty	39.0 (38.2–39.7)	35.3 (34.6–36.0)	6.9 (6.6–7.3)	8.5 (8.1–8.9)	1.5 (1.3–1.6)	3.3 (3.0–3.5)
Up to 2x threshold	32.7 (32.0–33.3)	28.7 (28.1–29.4)	5.4 (5.1–5.6)	4.8 (4.5–5.1)	1.0 (0.9–1.1)	3.3 (3.1–3.5)
>2x threshold	23.6 (23.3–24.0)	18.5 (18.2–18.8)	4.6 (4.5–4.8)	1.9 (1.8–2.0)	0.7 (0.6–0.8)	3.6 (3.4–3.7)
**Marital status**						
Married	20.8 (20.4–21.1)	16.1 (15.7–16.4)	3.6 (3.4–3.8)	2.0 (1.9–2.1)	0.6 (0.5–0.6)	3.2 (3.1–3.4)
Widowed/Divorced/ Separated	31.7 (31.0–32.4)	28.3 (27.6–29.0)	3.7 (3.5–4.0)	4.6 (4.3–4.9)	0.9 (0.8–1.1)	2.6 (2.4–2.8)
Never married	38.0 (37.6–38.5)	32.4 (31.9–32.8)	9.1 (8.9–9.4)	5.4 (5.2–5.6)	1.5 (1.4–1.6)	4.6 (4.4–4.8)

**Abbreviations:** AI/AN = American Indian or Alaska Native; CI = confidence interval; NSDUH = National Survey on Drug Use and Health.

* Persons who reported current (past 30-day) use of at least one of the five tobacco product types (cigarettes, cigars, roll-your-own tobacco, pipe, and smokeless tobacco) were considered to be current users of any tobacco product. Persons who had at least one missing response to any of the tobacco product use questions were excluded from the analysis (18, 0.5% of the AI/AN respondents). AI/AN population comprised persons who identified AI/AN as their only race/ethnicity. Non-AI/AN population comprised non-Hispanic White; non-Hispanic Black; non-Hispanic Native Hawaiian/other Pacific Islander; non-Hispanic Asian; non-Hispanic multirace; and Hispanic.

^†^ Prevalence significantly different from corresponding estimate among non-AI/AN population.

^§^ Estimates not presented because of relative standard error (RSE) ≥30%.

^¶^ Poverty level indicates a person’s family income relative to federal poverty level threshold. https://www.census.gov/data/tables/time-series/demo/income-poverty/historical-poverty-thresholds.html.

Among AI/ANs, the prevalence of current use of any tobacco product was 1.31 times higher among males than among females (Table 2). Compared with prevalence among persons aged ≥50 years, prevalence was higher among those aged 34–49 years (PR = 1.68); 26–34 years (PR = 1.79); and 18–25 years (PR = 1.88). By education attainment, prevalence was higher among persons with some college (PR = 2.07); a high school diploma (PR = 2.16); and less than a high school diploma (PR = 2.37) than among those with at least a college degree. Compared with prevalence among persons with annual family income ≥$75,000, prevalence was 1.55 times higher among those earning <$20,000. By poverty status, prevalence was higher among persons living at up to twice the federal poverty threshold (PR = 1.21) and in poverty (PR = 1.43) than among those living at more than twice the federal poverty threshold. Compared with those who were married, prevalence was 1.33 times higher among persons who were never married (all p<0.05).

**TABLE 2:** Disparities in current use of any tobacco product among American Indians/Alaska Natives — National Survey on Drug Use and Health, United States, 2010–2015

Characteristic	Current use of any tobacco product* (%)	Prevalence ratio^†^ (95% CI)
**Sex**		
Male	49.7	1.31 (1.14–1.52)
Female	37.8	Referent
**Age group (yrs)**		
18–25	55.6	1.88 (1.53–2.32)
26–34	53.0	1.79 (1.43–2.25)
35–49	49.7	1.68 (1.34–2.11)
≥50	29.6	Referent
**Education**		
<High school	49.8	2.37 (1.64–3.43)
High school graduate	45.3	2.16 (1.51–3.09)
Some college	43.5	2.07 (1.44–2.99)
≥College graduate	21.0	Referent
**Annual family income**		
<$20,000	50.3	1.55 (1.21–1.99)
$20,000–$49,999	41.2	1.27 (0.99–1.64)
$50,000–$74,999	40.6	1.25 (0.93–1.69)
≥$75,000	32.4	Referent
**Poverty level**		
Poverty	51.3	1.43 (1.19–1.70)
Up to 2x threshold	43.5	1.21 (1.00–1.46)
>2x threshold	36.0	Referent
**Marital status**		
Married	37.9	Referent
Widowed/Divorced/Separated	40.9	1.08 (0.87–1.34)
Never married	50.5	1.33 (1.14–1.56)

**Abbreviation:** CI = confidence interval.

* Persons who reported current (past 30-day) use of at least one of the five tobacco product types (cigarettes, cigars, roll-your-own tobacco, pipe, and smokeless tobacco) were considered to be current users of any tobacco product. Persons who had at least one missing response to any of the tobacco product use questions were excluded from the analysis (18, 0.5% of the AI/AN respondents).

^†^ Prevalence ratios were computed as regression coefficients, with the group with the lowest prevalence of any tobacco use serving as the referent.

AI/ANs had higher prevalence of any tobacco product use and cigarette smoking than any other individual racial/ethnic group (Figure). Prevalence of cigar smoking among AI/ANs was lower than among blacks, but higher than among Hispanics and Asians. Prevalence of roll-your-own tobacco and pipe use among AI/ANs was higher than among whites, blacks, Asians and Hispanics, and prevalence of smokeless tobacco use among AI/ANs was significantly higher than prevalence among all other racial/ethnic groups, with the exception of NHOPIs (all p<0.05).

**FIGURE:**
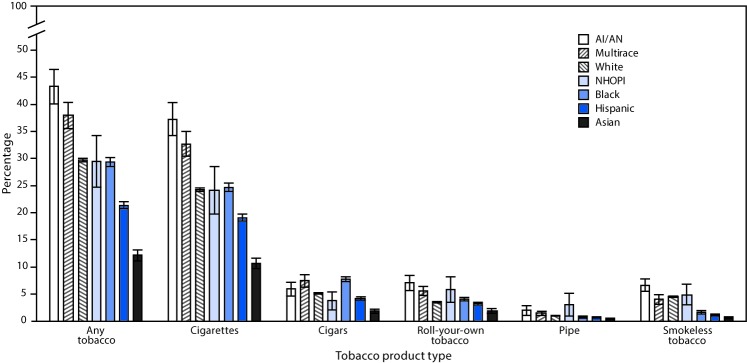
Prevalence of tobacco product* use by race/ethnicity^†^ — National Survey of Drug Use and Health, United States, 2010–2015 **Abbreviations:** AI/AN = American Indian or Alaska Native; NHOPI = Native Hawaiian or Other Pacific Islander. * Persons who reported current (past 30-day) use of at least one of the five tobacco product types (cigarettes, cigars, roll-your-own tobacco, pipe, and smokeless tobacco) were considered to be current users of any tobacco product. Cigars include big cigars, cigarillos, and little cigars. Smokeless tobacco includes snuff, dip, chewing, and snus. ^†^ AI/AN population comprised persons who identified AI/AN as their only race/ethnicity. Unless otherwise specified, all racial/ethnic groups are non-Hispanic.

## Discussion

During 2010–2015, the prevalence of current use of any tobacco product was significantly higher among AI/ANs than among non-AI/ANs, overall and among all assessed subgroups, except persons with at least a college degree. Among AI/ANs, the greatest disparity was associated with level of education: prevalence of any tobacco product use was 2.37 times higher among persons with less than high school diploma than among those with a college degree or higher. Socioeconomic status has a strong, inverse relationship with tobacco product use (*5*). Given that a higher percentage of AI/ANs live in poverty than do non-AI/ANs (28.4% versus 15.3% nationally) or have less than a high school education (23% versus 14% nationally),^§§^ addressing inequalities in education and poverty among AI/ANs might help reduce the high burden of tobacco product use among this population. Additional research is needed to identify the role of other factors (e.g., cultural, environmental, social) that might explain some of the observed differences.

Some American Indian tribes have long used traditional tobacco in cultural ceremonies of medicinal and spiritual importance (*6*). However, evidence suggests that commercial tobacco products, such as cigarettes and packaged loose tobacco, are being increasingly substituted for ceremonial purposes (*6**,**7*). In addition, tobacco products are less expensive on tribal lands, which might increase tobacco access and consumption (*8*). The tobacco industry has also been shown to target AI/ANs by marketing of cigarette brands with cultural icons, names, and symbols belonging exclusively to AI/ANs (*9*).

The equitable implementation of evidence-based tobacco control interventions, such as comprehensive smoke-free policies, is important to reduce tobacco product use among AI/ANs. CDC has implemented population-level strategies to help reduce disparities among AI/ANs, including Good Health and Wellness in Indian Country, an initiative that works to reduce commercial tobacco product use, while improving nutrition, physical activity, health literacy, and community-clinical linkages for AI/AN populations.^¶¶^ Moreover, CDC’s Tips From Former Smokers tobacco education campaign uses culturally appropriate mass media campaigns to warn about the health risks of smoking. Some of this work is tailored toward racial/ethnic minorities, including AI/ANs.*** Reducing disparities in use of tobacco products will require focusing more attention on populations carrying a disproportionate burden of tobacco product use and dependence, and increasing reach to such groups through efforts that directly affect the scope of services and facilities serving those populations.

The findings in this report are subject to at least four limitations. First, tobacco product use and other sociodemographic characteristics were self-reported and subject to recall and social desirability bias. Second, small sample sizes resulted in imprecise estimates that could not be reported for some sociodemographic subgroups. Third, data were unavailable for certain tobacco products, including electronic cigarettes and hookahs. Finally, these analyses used data pooled across multiple years, and therefore, do not reflect possible secular trends in tobacco product use.

Tobacco use is associated with cultural norms and socioeconomic factors such as education and poverty (*1*). Thus, culturally appropriate strategies are important when addressing tobacco-related disparities among AI/ANs (*9*). These strategies could include engaging traditional healers and respected community elders and fostering respect for traditional/ceremonial use of tobacco as a reason for not using tobacco recreationally,^†††^ while also addressing the social determinants of health (*10*). Creating partnerships within the AI/AN community might also help increase access to and use of evidence-based cessation resources.

**Summary**

**What is already known about this topic?**

Whereas significant progress has been made in reducing overall commercial tobacco product use, disparities persist, with American Indians/Alaska Natives (AI/ANs) having one of the highest cigarette smoking prevalences of all racial/ethnic groups.

**What is added by this report?**

Prevalence of current tobacco product use was significantly higher among AI/ANs than among non-AI/ANs for any tobacco product (43.3% versus 27.7%), cigarettes (37.3% versus 23.0%), roll-your-own tobacco (7.1% versus 3.5%), pipes (1.9% versus 0.9%), and smokeless tobacco (6.6% versus 3.5%). Among AI/ANs, prevalence of current use of any tobacco product was higher among males (49.7%), persons aged 18–25 years (55.6%), persons with less than a high school diploma (49.8%), persons with annual family income <$20,000 (50.3%), persons who lived below the poverty level (51.3%), and those who never married (50.5%).

**What are the implications for public health practice?**

Addressing the social determinants of health and providing evidence-based, population-level, and culturally appropriate tobacco control interventions could help reduce tobacco product use and disparities in tobacco product use among AI/ANs. Such interventions could include engaging native community leaders and fostering respect for traditional/ceremonial use of tobacco as a reason for not using tobacco recreationally.

## Footnotes

*
https://www.healthypeople.gov/ .

† Commercial tobacco is defined as tobacco that is manufactured by the tobacco industry for recreational use. http://keepitsacred.itcmi.org/tobacco-and-tradition/traditional-v-commercial/.

§ The survey weights were recalibrated by dividing by 6 (number of years pooled) to ensure that estimates were nationally representative.

¶ Until the 2014 survey, snus was not included in smokeless tobacco questions in NSDUH. It was first added in the 2015 survey.

** Respondents who had at least one missing response to any of the five tobacco product type questions were excluded from the analysis (752 [0.3%] respondents; 18 [0.5%] AI/AN respondents and 734 [0.3%] non-AI/AN respondents).

†† Poverty level was assessed since 2003. Poverty level indicates a person’s family income relative to federal poverty thresholds. https://www.census.gov/data/tables/time-series/demo/income-poverty/historical-poverty-thresholds.html.

§§
https://www.census.gov/newsroom/releases/archives/facts_for_features_special_editions/cb11-ff22.html .

¶¶
https://www.cdc.gov/chronicdisease/tribal/factsheet.htm .

†††
http://keepitsacred.itcmi.org/tobacco-and-tradition/traditional-v-commercial/ .
